# Resonant Anti-Reflection Metasurfaces for Infrared Transmission Optics

**DOI:** 10.1021/acs.nanolett.3c02375

**Published:** 2023-09-21

**Authors:** John Brewer, Sachin Kulkarni, Aaswath P. Raman

**Affiliations:** Department of Materials Science and Engineering, University of California, Los Angeles, Los Angeles, California 90095, United States of America

**Keywords:** metasurface, anti-reflection, Mie resonance, transmission enhancement

## Abstract

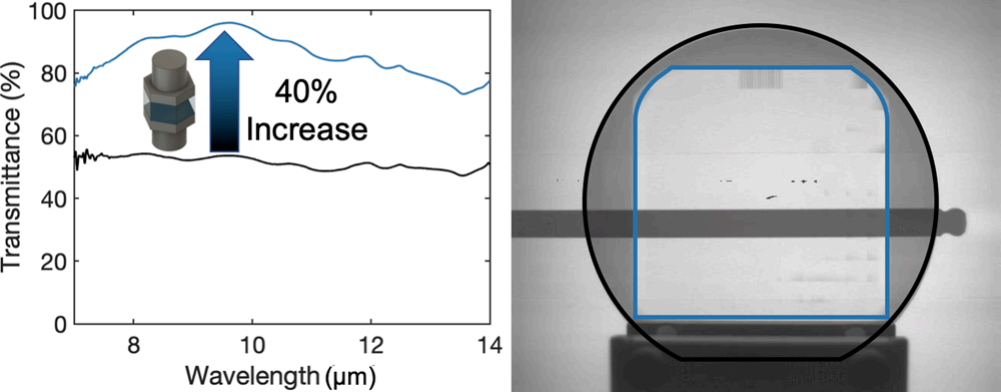

A fundamental capability needed for any transmissive optical component is anti-reflection, yet this capability can be challenging to achieve in a cost-effective manner over longer infrared wavelengths. We demonstrate that Mie-resonant photonic structures can enable high transmission through a high-index optical component, allowing it to function effectively over long-wavelength infrared wavelengths. Using silicon as a model system, we demonstrate a resonant metasurface that enables a window optic with transmission up to 40% greater than that of unpatterned Si. Imaging comparisons with unpatterned Si and off-the-shelf germanium optics are shown as well as modulation transfer function measurements, showing excellent performance and suitability for imaging applications. Our results show how resonant photonic structures can be used to improve optical transmission through high-index optical components and highlight their possible use in infrared imaging applications.

Increasing the transmission efficiency of optical components is broadly desirable in optical system design across all wavelengths. In the long-wave infrared band from 7 to 14 μm, for applications such as thermal imaging, high transmission efficiency is key for desirable performance due to the intensity-sensing nature of microbolometer-array-based detectors.^1,2^ Single-layer or multilayer thin film interference coatings are today’s standard for anti-reflection and transmission enhancement and are well developed.^3−5^ However, this approach requires materials specific coating stack engineering, which can be difficult and time-consuming depending on the system.^6−11^ This is because coating materials with a desirable refractive index and absorption over a given band may not exist and must be mechanically compatible with other stack materials. These difficulties are compounded by the desire for stacks that achieve some combination of durability, angular acceptance, and polarization insensitivity. Over longer wavelengths, thicknesses of the layers and the overall stack increase substantially, resulting in both added costs and limits to performance.

An alternative approach to anti-reflection is to use gradient index structures. To enable a gradual change in the refractive index along a depth dimension, gradient index structures typically employ depth modulated material-aggregate/sol–gel-based coatings or, alternatively, use so-called “moth-eye” anti-reflective structures. In the aggregate approach, nanoparticles are aggregated or created using processed chemical precursors, with sol–gels generally used to tailor particle densities at the interface in order to achieve a gradient index.^12−16^ Other recent methods have used spinodal separation methods followed by etching to create porous structures.^17,18^ In contrast, moth-eye geometries take advantage of subwavelength porous or high aspect ratio cone and pillar geometries which are fabricated by directly etching the surface of a substrate, and which can be treated as effective media used to minimize index mismatch between air and the higher-index substrate.^19−26^ While these approaches can compete with and exceed thin-film-coating-based approaches in terms of raw transmission, there is evidence that random structures can exhibit significant diffuse scattering depending on their geometry,^27,28^ which would cause reduced contrast when used for imaging. Additionally, the geometries of these structures often result in increased mechanical fragility and susceptibility to environmental contamination and abrasion, which can greatly reduce their performance over time in harsh environments.^29−32^

More recently, another approach to anti-reflection has emerged that uses resonant photonic structures at the interface between two media to decrease reflection. Unlike gradient-index-based approaches, resonant anti-reflection approaches use subwavelength photonic structures that are mechanically durable, robust, and easily fabricable. Early work investigated using metallic surface resonator elements which leveraged dipole resonances to achieve anti-reflection and preferentially forward scatter light.^33−35^ Later work introduced an alternative method employing all-dielectric Mie-resonant structures.^36−40^ This approach has primarily been explored over visible and UV wavelengths for absorption enhancement in solar cell applications. Early work from Spinelli et al.^36^ showed that monolithically coupled nanopillar structures exhibited scattering cross-sections up to 5 times larger than their geometrical cross-sections. This, in conjunction with the overlap of the resonant modes in the nanopillars with propagating modes of the substrate, results in a leaky mode that preferentially forward scatters into a high index material. The specific overlap of magnetic and electric dipole modes determines the resonance shape and is what allows the ensemble response to reproduce the incident wave into the substrate. The anti-reflection effect is enabled as a result of destructive interference between these electric and magnetic dipole modes,^41^ also referred to as the substrate-mediated Kerker effect.^42^ Theoretical conditions to maximize the effectiveness of this resonance-based approach have been established.^43^ Further developments enhanced the bandwidth of anti-reflection using multiresonant structures,^44^ as well as the previously discovered effective index support^45^ of Fabry–Perot resonances.^46^ While actively explored for solar absorption enhancement, to our knowledge, resonant anti-reflective approaches have not been demonstrated for transmission optics. Anti-reflection for imaging must enable high throughput and preservation of the incident wavefront through both high index interfaces. This capability, if possible to enable by the Mie-resonant approach, is of particular interest in long-wave infrared optical components, where anti-reflection via either conventional thin film or gradient index approaches can be challenging while meeting environmental and imaging-based constraints.

Motivated by these considerations, we propose and experimentally demonstrate a resonant anti-reflection approach to maximize transmission through an optical component while maintaining overall image quality. We design and optimize Mie-resonant nanophotonic structures and show through simulations that the overall phase front of incident light is preserved, meeting the key capability outlined above. We then fabricate the nanophotonic structures at the wafer scale on both sides of a double side polished (DSP) Silicon wafer, demonstrating strong anti-reflection and transmission over the LWIR wavelength band, with up to 40% transmission enhancement relative to unpatterned Si. We furthermore measure and calculate a modulation transfer function (MTF) of the Mie-resonant-structure-treated Si wafer and demonstrate image quality preservation, enabling utilization of this approach even for high-resolution imaging applications. Importantly, our approach shows equal or better transmittance than the previously mentioned coatings on Si at all investigated angles in the thermal band. Further, its transmittance performance is comparable to off-the-shelf broadband anti-reflection coated germanium alternatives at peak transmittance wavelength, enabling an alternative lower-cost material for window optics in LWIR and thermal imaging applications.

While Mie-resonant anti-reflection has been actively explored for absorption enhancement, there are important considerations that differentiate its utilization for imaging through transmission enhancement. Broadly, we group the goals of anti-reflection into three main categories, depicted in Figure 1. The first category is absorption enhancement, motivated by applications such as solar cells. Here, the back plane of the cell is typically assumed to be diffuse and fully reflective, and no light transmits through. Light is intended to be coupled into the media and absorbed as efficiently as possible to generate the greatest photocurrent. The second category corresponds to a transmissive back plane (such as in multijunction cells) which only considers maximization of optical power of a given band with no regard to image preservation. The final category considers the scenario in which power throughput is maximized while also ensuring image preservation. The concept of “image preservation” is meant to convey that the wavefront integrity is preserved and not degraded to a degree which makes resolving an object impossible. Our investigation focuses on this final case, which has not been explored in the LWIR range using a resonant-based approach to the best of our knowledge.

**Figure 1:**
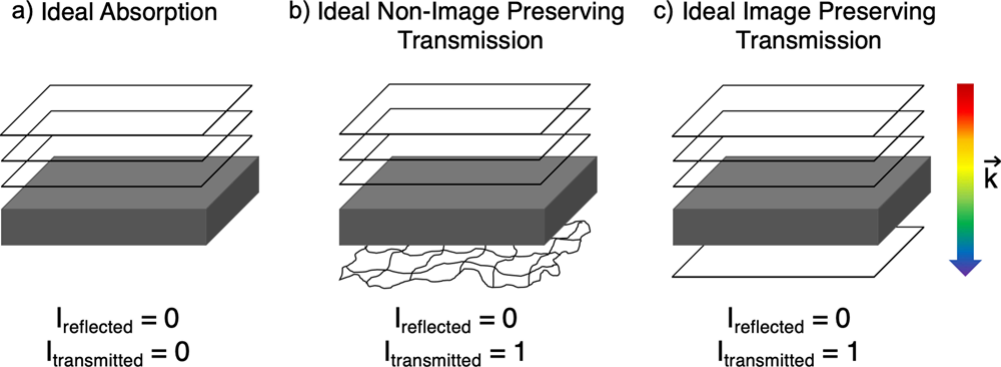
A schematic comparison of anti-reflection for the following: (a) Absorption enhancement: In this case, waves enter the substrate but do not leave it, being absorbed either as heat or generating photocurrent. As they are not exiting, their output state is disregarded. (b) Non-image preserving transmission enhancement: In this case, waves enter the substrate and are scattered by either or both of the front and rear surfaces. This configuration could occur in a multijunction cell, or any diffuser optic. (c) Image preserving transmission enhancement: In this case, the shape of the incoming wave is preserved as it exits the substrate optic. Input plane waves exit as plane waves, and waves with more complex wave fronts will be maintained with an added phase due to propagation.

Dielectric Mie resonators can induce excellent anti-reflection through the unique forward scattering phenomena enabled by an ensemble response of individual resonators. In our case, a subwavelength periodic array of resonators causes enhanced transmission through the interfaces of Si at the relevant LWIR wavelengths which maintains the integrity of the overall input wavefront. This approach is in principle highly broadband and omnidirectional and has minimal polarization sensitivity, all of which are desirable properties for an imaging optic. Additionally, the approach is entirely binary, enabling fabrication by conventional photolithographic means. We note that going against conventional Fresnel-law-based intuition, the all-dielectric Mie-resonant approach relies on a *large* index contrast in order to produce high quality *suppression* of reflections. Optical materials generally used for transmission optics in the LWIR wavelengths, such as Ge and, as we will demonstrate, Si, have suitably high refractive indices. This presents a unique opportunity to use the resonant approach to increase transmission efficiency in these wavelengths despite the large index contrast normally resulting in a poorer reflective performance.

We opted to use Si as our material system due its wide availability, fabrication maturity, and relatively low overall transmittance in the LWIR^47,48^ (∼50% through a 500 μm thick bare substrate over the full λ = 7–14 μm band) which allows for significant and easily noticeable performance improvement. We applied the resonant approach to arguably the simplest optical component, a window. Window optics provide a clear and useful proof-of-concept platform for our approach. They are ubiquitous for protection of sensitive components in optical systems from the environment and often necessary to prevent ingress of dust and humidity to the rest of a lens column. As noted above, Si’s relatively high intrinsic absorptivity in the LWIR means that it is not typically used for imaging or window optics in the face of higher transmission Ge, ZnSe, or ZnS alternatives. We note however, that large thicknesses may not be essential in fulfilling a window’s protective functionality (i.e., when impact resistance or high pressure tolerance is not a necessary function of the window). In cases where imaging is desirable, more recent advances in metasurface design demonstrate that thin and flat focusing optical systems are possible, limiting the effects of Si’s intrinsic absorptivity on optical performance. Additionally, Si does hold notable mechanical advantages over the previously mentioned materials, having a lower density and higher hardness.^49−53^ Finally, there is evidence to suggest that in high temperature ambient environments, the absorptivity of Ge may exceed Si at thermal wavelengths,^54,55^ making Si an even more viable alternative in high temperature operation.

We first numerically investigated and optimized resonant anti-reflective designs scaled for Si’s index and the LWIR wavelength range, evaluating a range of different lattice and feature geometries assuming 2 identically patterned interfaces. We simulated the designs using rigorous coupled-wave analysis (RCWA) while assuming fabricable critical feature sizes based on available tooling. To facilitate optimization, we developed a custom figure of merit shown as eq 1 which rewarded integrated spectral transmittance through the optic while inflicting a severe penalty on unfabricable designs:
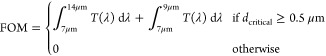
1

The result of this optimization, shown schematically in Figure 2a, yielded the best performance of the designs explored within typical wafer-scale fabrication limits. We note here that the added 7–9 μm integral term sought to reward higher transmission at more energetic wavelengths, but in principle, resonator geometries could be tailored to move the peak to longer wavelengths as well. *E*-field component plots for a demonstrative 16 μm thick Si substrate are shown in Figure 2b, which show the aspect of our design that is of particular importance to our application: the coherent character of the forward scattered waves through the interface, resulting from the overlap of electric and magnetic dipole resonance overlaps present. Further field plots to demonstrate the resonance shape and dipole overlap can be found in the Supporting Information. This phenomenon occurs when the waves are scattered into and out of the patterned high index media. Simulated spectral transmission comparisons are shown in Figure 2c, highlighting the transmission performance increase we expected from our approach.

**Figure 2:**
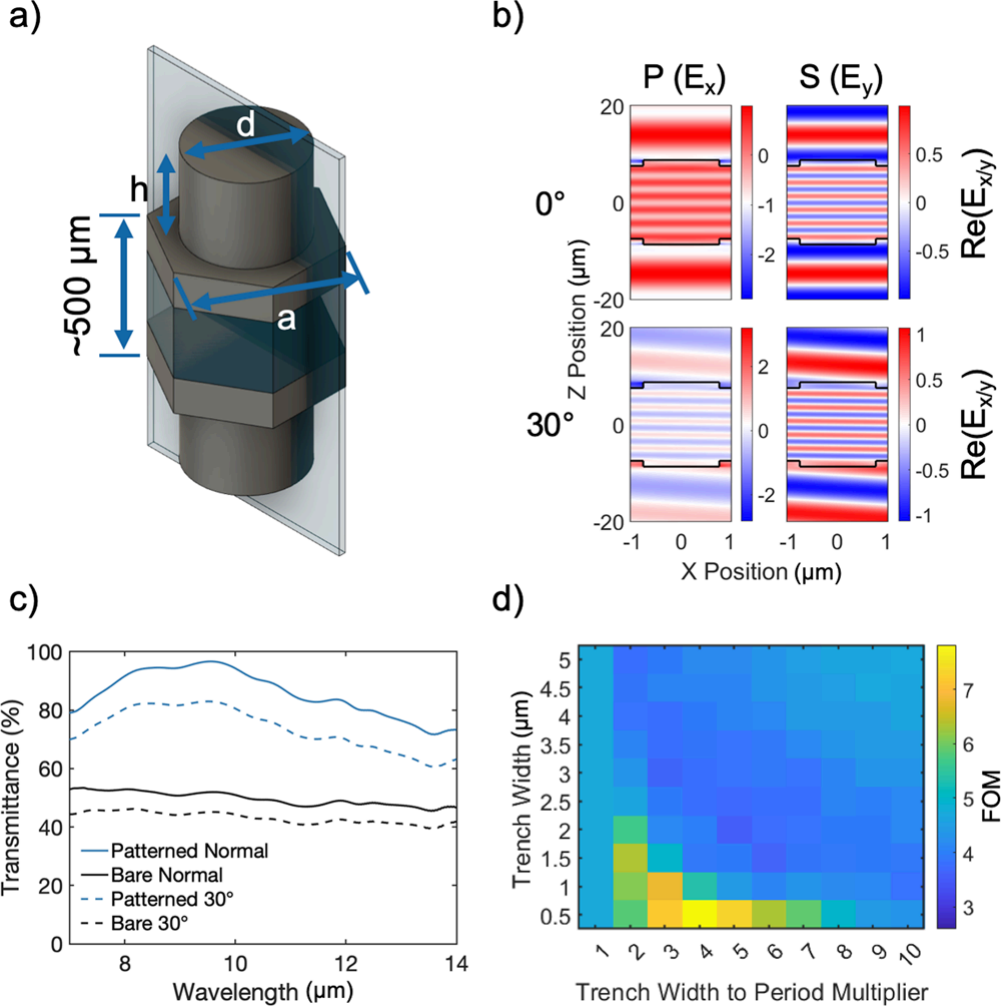
(a) Pictorial schematic of a single hexagonal unit cell of the finalized design. The design is patterned on both sides of the wafer. *d* = 1.5 μm, *h* = 1.2 μm, *a* = 2.0 μm, resulting in a critical trench feature size of 0.5 μm. The plane cross-section through the unit cell depicted in part b is shown. (b) *E*-field component plots for normal and 30° incidence. Simulation cross-section at 9.6 μm for a truncated 16 μm thick substrate to demonstrate plane-wave propagation, shown for P and S polarizations. (c) Comparison of simulated spectral transmission for unpolarized light at normal and 30° incidence. There is at least a 20% uplift over the entire band and 0°–30° angular range. (d) Slice of the simulation parameter sweep for optimal ∼1.2 μm cylindrical pillar feature height, showing trench width vs period multiplier. The period for a given tile is calculated by multiplying *x* and *y* positions for that tile. Feature width can then be calculated by subtracting the *y* value from the resulting period.

We highlight a portion of the optimization landscape in Figure 2d, which shows a slice of the 3D optimization (feature height, period, and feature width) at a feature height of 1.2 μm. The *y* axis shows the trench width between each resonant element (the critical feature in terms of fabrication), while the *x* axis shows the multiplier of the trench width to determine the period. The period for a tile is then given by multiplying the *x* and *y* axis values for that tile. This allows scanning a large number of features while ensuring the prevention of unphysical or unfabricable designs.

We fabricated the optimal design on float zone process grown 500 μm thick double side polished intrinsic Si substrates. We lithographically patterned the optimized Mie-resonant photonic structures of hexagonally packed cylindrical pillars with a periodicity of 2 μm, a height of 1.2 μm, and a diameter of 1.5 μm onto both sides of the wafers (see the Supporting Information). A high magnification SEM image of the design tilted at 35° is shown in Figure 3a showing good consistency and overall design fidelity.

**Figure 3:**
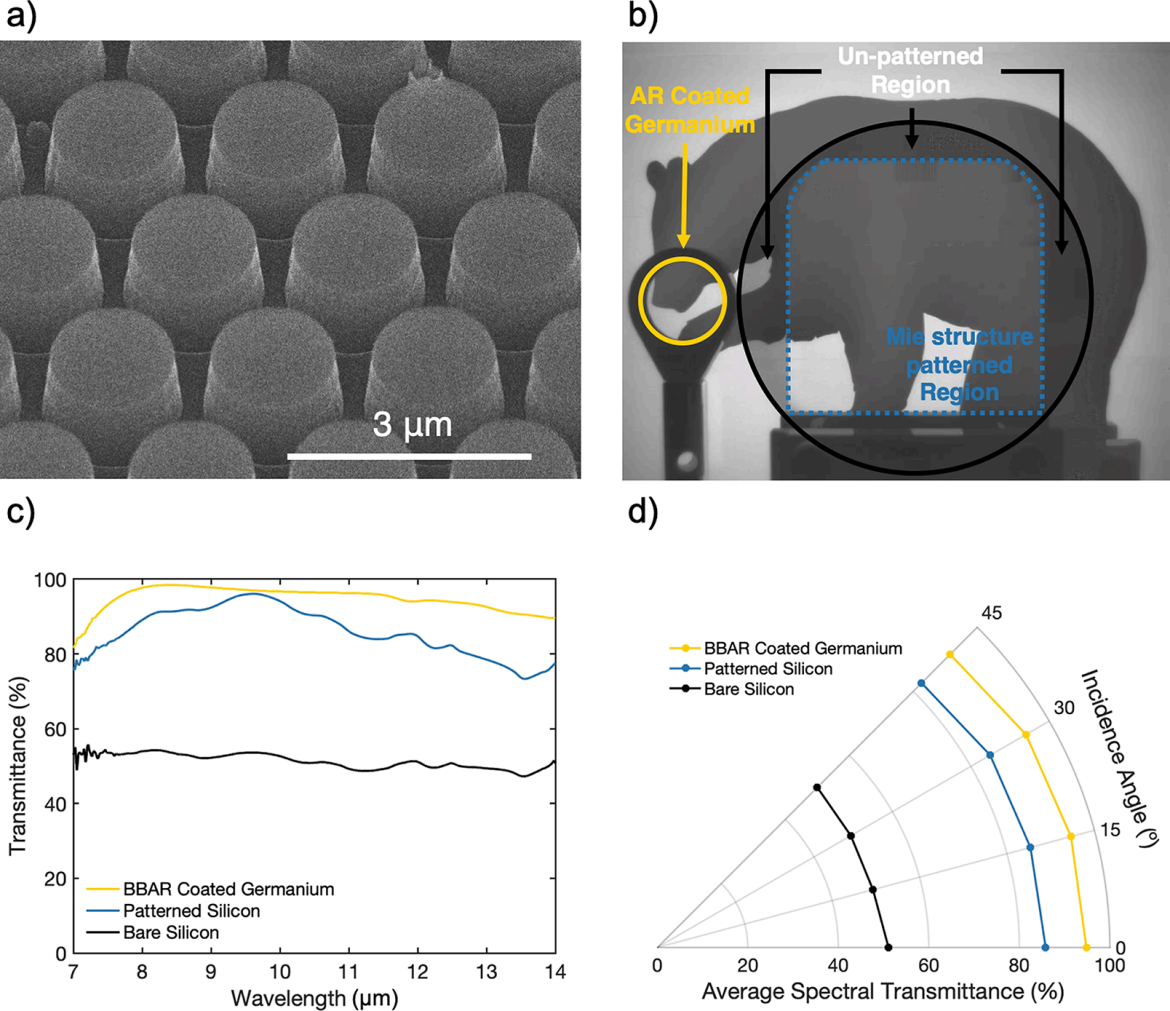
(a) SEM image of surface patterning, which is present on both sides of the substrate. (b) Imaging test of the fabricated optic taken on an FLIR BOSON+ thermal camera. Borders to the patterned region and wafer edge have been added for clarity. Si transmission improvement is immediately noticeable compared to unpatterned edges of wafer and compares favorably with the Ge window on the left. (c) Comparison of unpatterned (black) and patterned (blue) intrinsic Si and AR coated Ge (yellow) windows at 0° incidence. Patterning results in an up to 40% increase in transmission over the bare case. (d) Angular falloff plot showing integrated spectral transmission over the 7–14 μm band for bare Si, BBAR coated Ge window, and Mie patterned Si.

LWIR spectral measurements of the device can be seen in Figure 3b, comparing the performance of a bare Si wafer, a patterned Si wafer, and a 1 mm thick Ge optic. As the design is highly periodic, diffraction is a possible concern in terms of imaging, but we note that the patterned features are small enough with respect to the 7–14 μm band that they fall outside the diffractive regime for all incidence angles ( for the entire band). This lack of diffractive behavior is observable in the *E*-field component plots at each polarization for 2 cross-sectional cuts of the unit cell in Figure 2b, showing planar propagation of the waves in either polarization and at angular incidence. Finally, as the window optic will be used in an imaging system, its transmission spectra are only a proxy for its true performance. In reality, if the surface relief structure causes undesirable scattering-based effects, these will manifest only when imaging through the device. As an initial test, an image of a thermal target was taken through the patterned device, as shown in Figure 3c. The square patterned area has a dotted outline, with wafer segments outside of this area being unpatterned. A Ge LWIR AR coated window is also shown as a comparison, with areas outside both window regions acting as a control. A clear difference in transmission can be seen between these areas, while imaging integrity is maintained.

As a final, more quantitative demonstration of imaging performance, we performed a tangential broadband modulation transfer function (MTF) measurement using a slanted edge target through our patterned Si optic, an AR coated Ge window, and no window. Images were then taken at 0° (on-axis), 10° (70% field), and 14° (full-field) field angles. While the full field of view is around 15°, enough of each side of the slant target needed to be visible in the image for proper measurement. Analysis and MTF calculation was done using sfrmat5, a publically available code used for MTF measurement of systems using slant edge targets.^56^ Comparisons at each field angle are shown in Figure 4, showing that the MTF is comparable between our photonic Si optic, the commercial Ge optic, and the case without a window at all 3 field angles. We note that, while this measurement was performed in as controlled a manner as possible with available equipment, it is primarily meant to demonstrate the comparison of our optic to notable alternatives and is not intended to serve as a specification for any of the individual optics or complete system under test. MTF is highly dependent on both the imaging system and the detector used, and acceptable values of MTF are highly application- and context-dependent. In our case, we have used the same detector and focusing lens column in all measurements, with the only changes being the addition of the different window optics in order to produce a useful comparison between these window components specifically and not all possible optical systems. Additionally, while the response for each case is close, Figure 4b’s inset shows that the MTF with window optics added is in general higher than without. We attribute this to the frequency filtering of the windows, preferentially rejecting poorly corrected source wavelengths from entering the rest of the imaging system. These relative positions hold at 97% of the plotted points. Differences at each measured spatial frequency point for all three field angles can be found in the Supporting Information. The maximum effect with the added window over all plotted frequency points and angles only amounts to a difference of 3.6% in the modulation factor.

**Figure 4:**
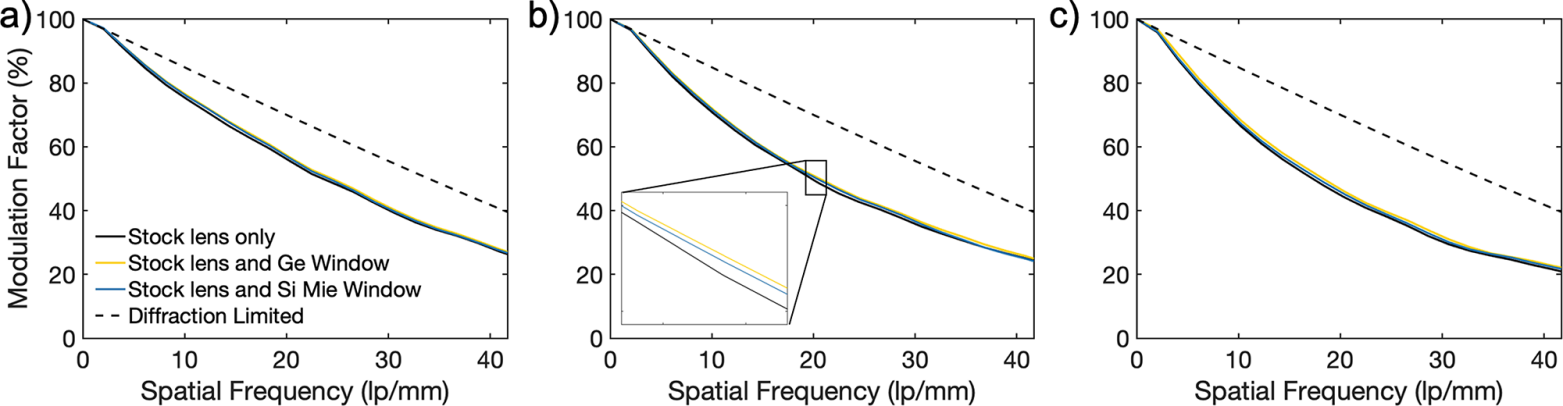
Modulation transfer function (MTF) comparison between the control LWIR optical system, Ge window optic, and Mie-resonant high transmission Si. (a) On-axis, (b) at 10° or 70% field, and (c) at 14° or 93% horizontal field. MTF values are comparable between all 3 systems at all field angles, indicating that scattering from the surface does not cause significant imaging performance loss. To highlight the relative magnitudes of the curves more clearly, a zoomed inset has been included in part b, reflecting that the MTF presents as higher with additional optics, which we note is likely an artifact resulting from spectral filtering occurring from the added optics. The camera used was a FLIR BOSON+, 640 × 512 pixel camera with 12 μm pixel pitch. Spatial frequency data was plotted out to a detector Nyquist frequency of 41.6 lp/mm.

The Mie-resonance-based approach we have demonstrated here offers several possible advantages over current state-of-the-art thin film coatings at these wavelengths. While the literature investigating AR coatings for Si over LWIR wavelengths is scarce, known thin film approaches use ZnS^60^ or a combination of ZnS and YF_3_ as the coating material, with an Al_2_O_3_^57^ or MgO^58^ adhesion layer, respectively. While thin film adhesion and film stress are generally sensitive to growth conditions in most materials systems, highly optimized many-layer coatings with these materials have suffered from delamination issues which have not been solved.^58^ These film thicknesses were generally on the order of 1–2 μm. Additionally, thermal cycling has the ability to aggravate these issues in more complex systems.^59^ With these considerations in mind, although lithographic fabrication of Mie-resonant structures involves more complex processing, our AR approach holds promise for a variety of applications where thermal cycling durability, adhesion, scratch resistance, or high temperature performance are desirable. Moreover, several optics can be produced from a single wafer, and it is simple to engineer our solution and tailor its peak wavelength over the thermal band, for which a given substrate material is transparent. Compared to graded-index approaches, a Mie-resonant approach generally has similar fabrication difficulty and is less susceptible to dust, humidity, abrasion, and mechanical wear affecting its performance due to its lower aspect ratio. A cost comparison of these approaches is hard to estimate as materials prices, tool time, and inputs can vary greatly, but purely from the standpoint of substrate materials, even float zone Si is significantly less expensive than equivalent Ge substrates (see the Supporting Information).

In conclusion, we have demonstrated a method based on resonant forward-scattering microstructures to increase transmission in Si optical components over LWIR wavelengths. We show a performance increase of up to 40% with comparable performance to Ge at shorter wavelengths, where the optical power incident on the detector is greatest. We note that our approach shows equal or better performance than previously reported thin film coatings for Si at all shown angles. An intriguing future possibility exists in combining our resonant metasurface approach with focusing metasurface patterns on the opposing side of the same substrate to allow the use of Si LWIR optics, making it a viable alternative material platform in systems where throughput requirements are not as stringent. Additionally, the use of the Si in this configuration suggests intriguing possibilities for its use in active optoelectronic systems over long-wave infrared wavelengths.

## Supporting Information Available

Additional information on detailed fabrication methods, additional field plot data, additional angular spectra, AR approach comparisons, and experimental setup (PDF)
