# Supplementary material for: Resonant Anti-Reflection Metasurfaces for Infrared Transmission Optics
Source: Nano Lett. 2023 Sep 21;23(19):8940–6. doi: 10.1021/acs.nanolett.3c02375 (PMC10571145; doi:10.1021/acs.nanolett.3c02375)
Supplement: Supplementary file 1 — nl3c02375_si_001.pdf [file nl3c02375_si_001.pdf]

Resonant Anti-Reflection Metasurface for Infrared Transmission Optics (Supporting Information)

John Brewer, Sachin Kulkarni, and Aaswath P. Raman*

Department of Materials Science and Engineering, University of California, Los Angeles, Los Angeles, CA 90095, United States of America

E-mail: aaswath@ucla.edu

Materials and Methods

Initial fabrication was performed on 4” double side polished 500μm thick N-type silicon, which we found to be completely opaque in the infrared range using FTIR transmission measurements. These initial wafers were used to determine process flow and gain insight into how fabrication should best proceed, as well as to determine correct etch times for desired feature depth, and determine resist and anti-reflection curing and exposure properties. Fabrication proceeded onto P-type wafers, which were significantly less absorptive than purchased n-type stock and allowed determination of how fabricated devices differed from simulated geometries in spectral response. Final device fabrication was then performed on 4” double side polished 500μm thick intrinsic float zone silicon. Wafers were thoroughly cleaned out of pack in 30 minute 100$°C$ Piranha etch bath, then spin rinse dried. After cleaning, approximately 4000 Å of wet thermal oxide was grown on wafers for use as an oxide hard mask. The first side of the oxidized wafers were then processed on an SVG 8800 track coater. On track coater, wafers were HMDS vapor primed, then spin coated with AZ BARLI ii back side anti-reflection coating at 3 krpm, followed by a soft bake at 200$°C$ for 60 seconds. Wafers were then put back through track coater and coated with AZ MIR 701 positive tone photoresist at 5 krpm, followed by an automatic edge bead removal step. Wafers were then soft baked again at 90$°C$ for 90 seconds.

After photoresist was applied, exposure was done on an ASML PAS 5500/200 projection exposure stepper alignment tool. Mask pattern was designed such that field could be tiled continuously across wafers with multiple adjacent exposures, though per-field alignment was not perfect, and adjacent fields generally had small misalignments. Exposed wafers were then post exposure baked at 90$°C$ for 90 seconds. After post exposure bake, wafers were beaker developed in AZ 300 MIF developer for 60 seconds, then placed into a cascade bubbler rinse for 1 minute. Rinse was followed by a nitrogen gun dry, then hard bake at 118$°C$ for 60 seconds.

After hard bake, wafers were then RIE fluorine etched for hard mask release. Wafers

were then cleaned in a Matrix 105 oxygen plasma asher. Following plasma clean, residual photoresist was then cleaned off using 3:1 Piranha etch for 30 minutes. Wafer was then chlorine etched to desired depth in a PlasmaTherm SLR 770 ICP RIE tool.

Single side etched wafers were then taken through the above process again for the back side. After back side chlorine etch was completed, oxide hard mask present on both sides was etched away in HF dip, followed by a cascade bubbler rinse and nitrogen gun dry to complete the device. Metrology on initial fabrication attempts was done on a combination of Dektak contact profilometry and SEM image analysis to understand and characterize resist mask, hard mask, and etch depth steps. After device was completed, its spectra was measured using a Bruker INVENIO R FTIR tool to determine its IR transmission properties. IR image capture setup is described in the following section in detail.

Off-the-self Germanium window optic was a 1 mm thick 1" BBAR coated and purchased from Edmund Optics.

Supplemental Field Plots

Vector field plots of all E and H field components real parts at normal incidence are shown in Figure S1. Transverse cross-sections are taken at half the height of resonator feature, approximately 0.6 μm below top of micro-cylinder. Dipole resonance overlap is visible in the rightmost column of Figure S1, demonstrating the implicated phenomena which causes Mie-resonant AR behavior.

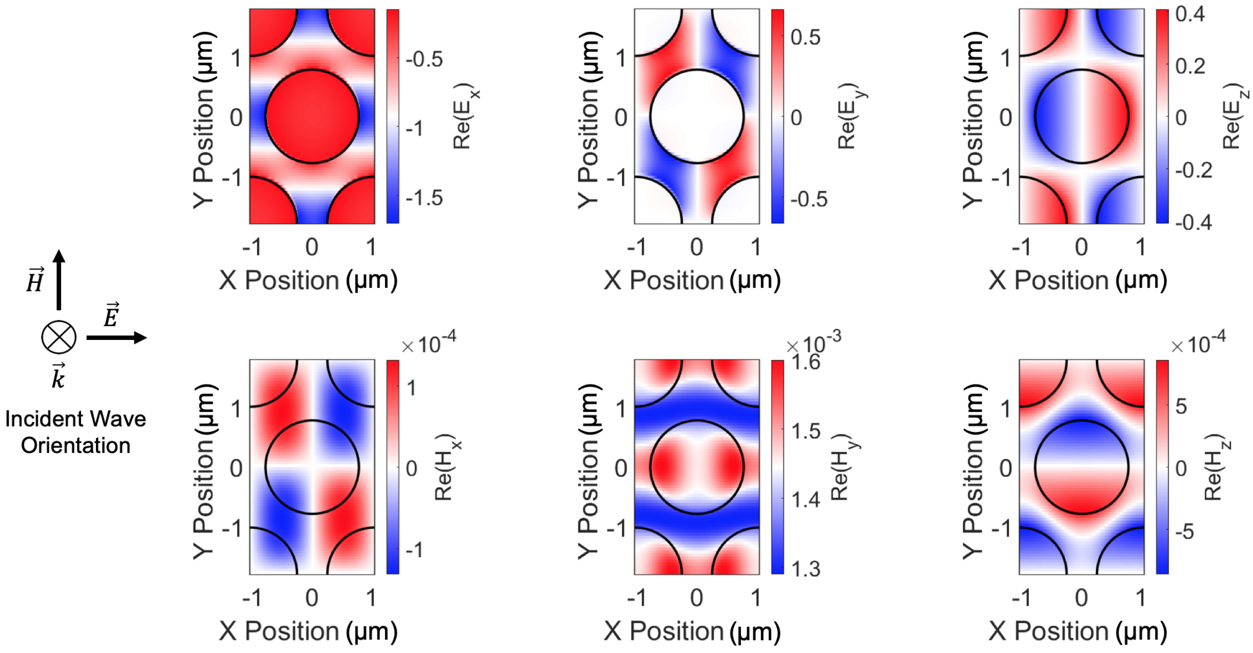

Figure S1: Tranverse plane field plots of Mie-Resonant AR structure at normal incidence, with structure boundaries indicated by black circles. E_z and H_z dipole resonances overlaps are visible in the rightmost column. Note that each plot has been range normalized to show greatest contrast.

Experimental setup of MTF Measurement

Image testing was done with FLIR BOSON+ thermal imager with 640 x 512 imaging resolution. Pixel elements have $12\mu m$ pixel pitch. Cardboard enclosure was used to block stray heat and light from lab environment from reaching detector body, and cardboard slats were used to prevent stray reflection of source light from optical table surface. Camera unit was placed approximately 2' from slant edge object which consisted of a piece of aluminum sheet metal stock placed in front of a vertically oriented hot plate surface, with an approximately $5.5°$ tilt applied along the factory cut edge. Aluminum object was slightly tilted with respect to normal camera incidence to prevent camera thermal signature (reflection) from affecting data collection. Hot plate was set to $100°C$ and allowed to thermally stabilize for 10 minutes before beginning image capture. High absorptivity aluminum tape was adhered to surface of hot plate to slightly flatten image field and give more greybody like response and increase target contrast. Camera was set to capture raw 16 bit .TIFF format images without software gain applied, and automatic flat field correction was turned off for image capture. For each field and device, the field was first manually flattened using software command, then a series of 10 images was taken. Images were cropped to the identical pixel locations, then cropped image counts were averaged and rounded to nearest integer count value. This final averaged image was then used to calculate MTF data. To determine imager "dark" count, a 2" x 2" piece of dry ice was imaged $< 1\ cm$ from imager lens body. This dark count value was then subtracted from all averaged devices taken for MTF data to give a "true" count value, and therefore modulation factor value.

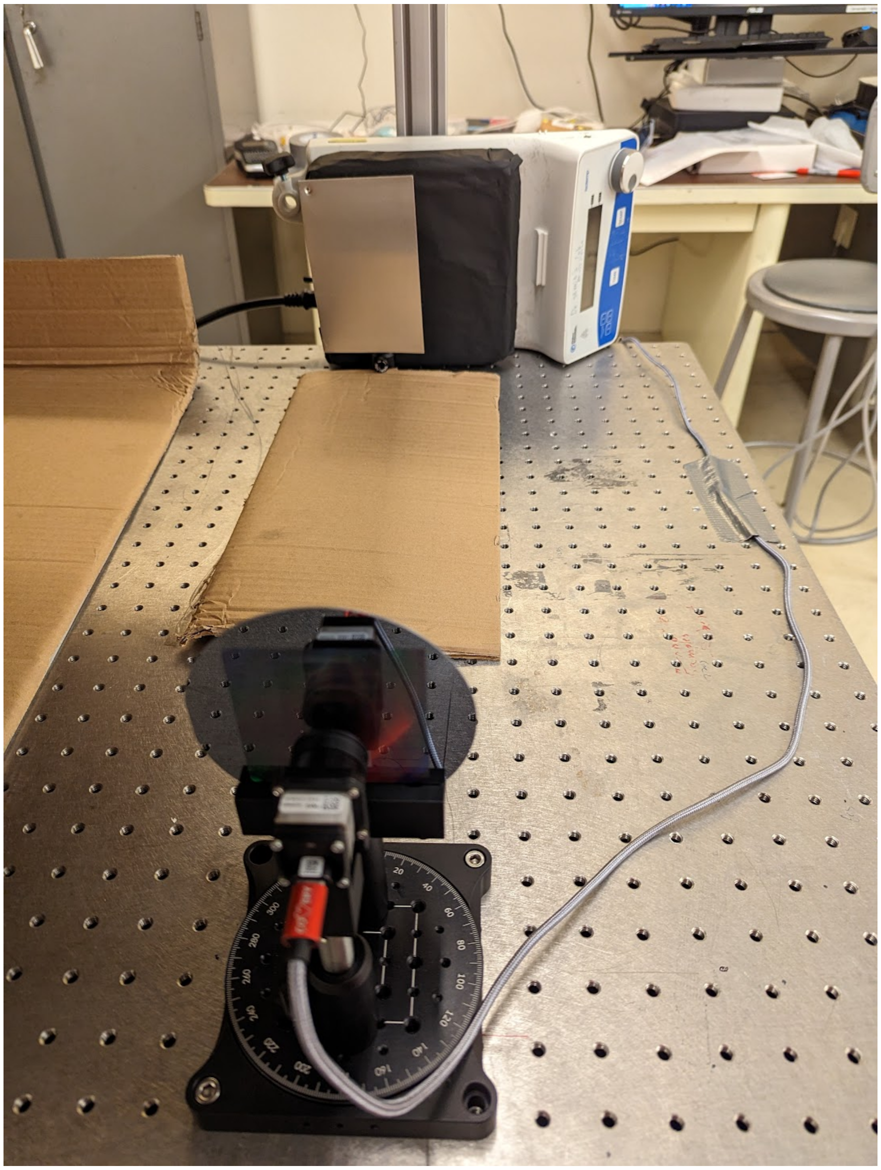

Figure S2: Picture of experimental setup. Camera and optic rotate together on stage for MTF measurements, to simulate window being a part of lens optical stack.

MTF difference between system with windows and stock system

To demonstrate that the inset figure shown in Figure 4b of the main text is accurate at nearly all spatial frequencies over all tested field angles, a difference plot is given Figure S3. The only exception to this occurs past 34 lp/mm at the 10 degree field angle, at which point our silicon optic performs slightly worse than the stock system. These points account for only 3% of those plotted. We note that the plots in Figure S3 are given in absolute % differences, that is, the raw modulation factors subtracted from each other. They are not "percent differences" in the proportional sense.

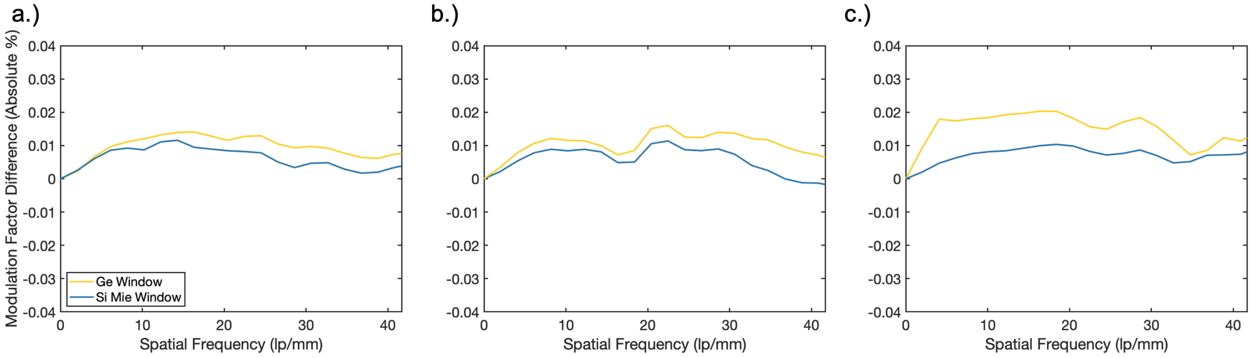

Figure S3: Difference between system with added Ge window and stock system (yellow) and system with Si window added and stock system (blue) at each measured spatial frequency a) On axis b.) at 10 degrees and c.) at 14 degrees.

Angular Spectra Comparison

Integrated spectrally averaged transmittance data is shown in the main text to clearly summarize results, but direct comparison of spectra can be seen in Figure S4.

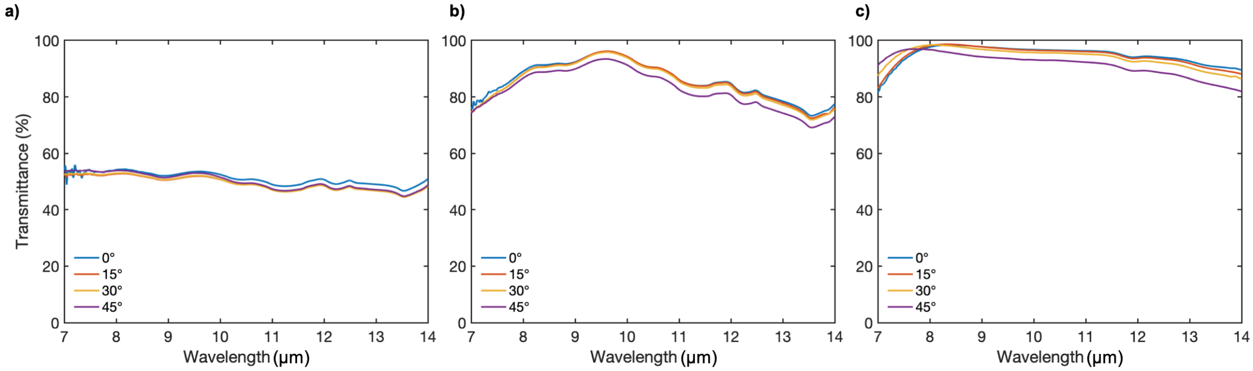

Figure S4: Angular spectra measurements for a)bare silicon b)Mie-resonant silicon and c) BBAR coated Germanium window optic.

Front to Back Pattern Misalignment Study

Due to the two-sided nature of the photonic structures, it is reasonable to believe that alignment between the patterning of the two surfaces is an important factor in the performance of the device. Due to the sub-wavelength nature of the features, misalignment generally results in no difference in performance. To confirm this, a series of simulations was run with the Mie-resonant patterns at different percentages out of phase at several incident angles and polarizations, shown in Figure S5. They show that at all but the highest angles, alignment of the two surfaces makes no difference. Even at 60°, the divergence seen for any amount of misalignment ends at around 7.5 um. The maximum effect occurs at 7um, which results in transmittance decrease of 7% at that wavelength, tapering off to 0% at 7.5um.

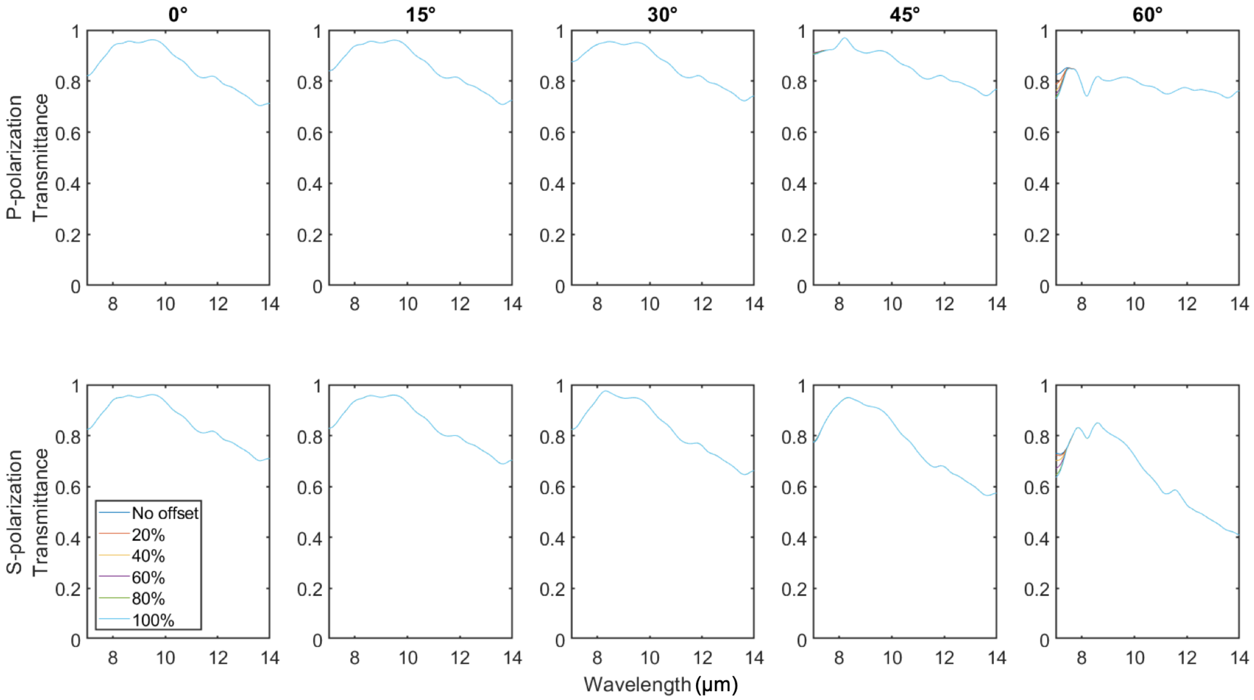

Figure S5: Angular spectra measurements for varying degrees of misalignment at 0°, 15°, 30°, 45°, and 60° incidence angles (columns) for S and P polarizations (rows). Note that all lines are overlapping, so only fully out of phase misalignment color is visible. This is confirmed by the 60° simulations, which show slight divergence at low wavelengths.

Qualitative comparison of AR approaches and materials

Below we present two tables qualitatively comparing the approaches and materials we cover within the work. While not meant to be comprehensive, this comparison aims to covers notable aspects of the AR approaches used for a functional window optic.

Table S1: A brief qualitative comparison table for the AR approaches covered within the work. Asterisk denotes that under normal conditions adhesion is very good, but large thermal swings can cause delamination, as covered in the main text.

	Thin Film	Gradient Index/Motheye	Mie Resonant
Cost	$	$$	$$
Performance	Very Good	Very Good	Very good
Fabrication Difficulty	Simple	Medium to intensive	Medium to intensive
Fabrication Scalability	Very Good	Medium	Medium
Scratch Resistance	Poor	Poor	Good
Soiling Resistance	Good	Poor	Good
Coating Adhesion	Very Good*	Very Good to monolithic	Monolithic

Table S2: A brief qualitative comparison table for the material systems covered within the work.

	Germanium	Chalcogenides	Silicon
Cost	$$$	$$$	$
Refractive Index	≈ 4	≈ 2 – 2.4	≈ 3.4
Absorption at 300K	Negligible	Negligible	κ ≈ 2E-5 to 3E-4
Hardness	Knoop 780	Knoop 120 - 240	Knoop 1150
Operating Temperature	Small bandgap means free carrier induced absorption becomes non-negligible @ > 65°C	Useable past 100°C	Useable past 100°C
Fabrication Difficulty	Simple	Medium	Simple
Tooling Maturity	High	High	High
